# Determining the Size of Representative Volume Elements for a Two-Dimensional Random Aggregate Numerical Model of Asphalt Mortar without Damage

**DOI:** 10.3390/ma17143387

**Published:** 2024-07-09

**Authors:** Sheng Liang, Jing Tao, Xiaoming Zhao, Zhong Liu, Derun Zhang, Chongzhi Tu

**Affiliations:** 1 School of Civil and Hydraulic Engineering, Huazhong University of Science and Technology, Wuhan 430074, China; sheng_liang@whut.edu.cn (S.L.); derunzhang@hust.edu.cn (D.Z.); 2 Shenzhen Road & Bridge Group Co., Ltd., Shenzhen 518001, China; taojing@szlqjt.com (J.T.); zhaoxiaoming-5@163.com (X.Z.); liuzhong@szlqjt.com (Z.L.); 3 School of Civil Engineering and Architecture, Wuyi University, Jiangmen 529000, China

**Keywords:** asphalt mortar, two-dimensional random aggregate model, representing volume elements, geometric analysis, numerical analysis

## Abstract

The size of the representative volume element (RVE) for the two-dimensional (2D) random aggregate numerical model of asphalt mortar in a non-destructive state, which directly affects the time required to simulate the linear viscoelastic behavior from asphalt mastic to asphalt mortar. However, in the existing literature, limited research has been conducted on the size determination of the numerical model RVE for asphalt mortar. To provide a recommended size for the typical 2D random aggregate numerical model RVE of asphalt mortar in a nondestructive state, this paper first applies the virtual specimen manufacturing method of asphalt concrete 2D random aggregate to asphalt mortar. Then, it generates numerical model RVEs of asphalt mortar with different maximum particle sizes, after which geometric and numerical analyses are conducted on these models. Finally, based on the geometric and numerical analysis results, the recommended minimum sizes of RVE for the 2D asphalt mortar numerical model are provided.

## 1. Introduction

**Figure 1:**
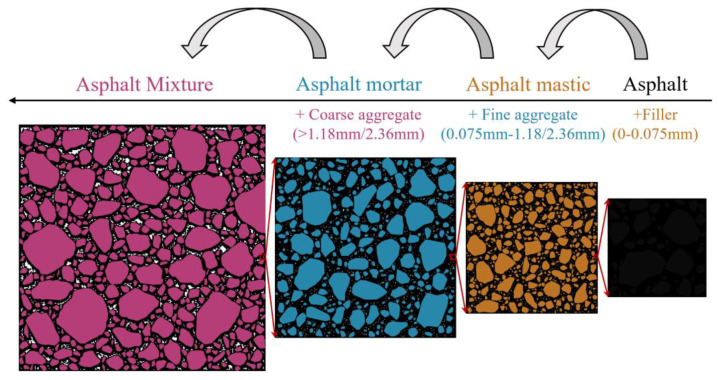
The four scales of asphalt mixture.

Asphalt mixtures can be divided into four scales: asphalt, asphalt mastic, asphalt mortar, and asphalt mixtures [1,2,3,4]. The relationship of those four scales is shown in Figure 1, in which the asphalt mixture is composed of coarse aggregate and asphalt mortar [5,6], the asphalt mortar is composed of fine aggregate and asphalt mastic [7], and the asphalt mastic is composed of filler and asphalt [8]. Exploring the interrelationships between these four scales is an important component of the digital design of asphalt mixtures. In order to explore the interrelationships between the components of asphalt mixtures, in recent years, many researchers have introduced numerical simulation methods into the cross-scale analysis of the linear viscoelastic behavior of asphalt mixtures and have achieved some results [9,10,11]. However, most of these studies have focused on the cross-scale simulation from asphalt mortar to asphalt mixture, and there are few cross-scale simulations from asphalt mastic to asphalt mortar, which to some extent limits the development of digital design of asphalt mixtures. To further promote the development of digital design of asphalt mixtures, it is necessary to conduct cross-scale simulation research on the relationship of asphalt mastic to asphalt mortar.

In cross-scale simulations, representative volume element (RVE) is a carrier that connects materials of different scales [12,13], and its size is closely related to the computational efficiency of a simulation [14]. Reducing the size of RVE as much as possible while ensuring simulation accuracy is an effective way to improve the computational efficiency of a cross-scale simulation [15]. Therefore, before conducting cross-scale simulations of the linear viscoelastic behavior from asphalt mastic to asphalt mortar, it is necessary to determine the size of the RVE of the numerical model of asphalt mortar in a nondestructive state. The process of determining the size of the asphalt mortar numerical model RVE in a nondestructive state can be divided into two steps: (1) manufacturing different sizes of asphalt mortar numerical model RVEs, and (2) analyzing the characteristics of different sizes of RVE and determining the minimum size. Below is a summary of the existing research corresponding to these two steps.

First, in terms of manufacturing the numerical model RVE for asphalt mortar, the development of virtual specimen methods has been conducted by researchers in their attempts to extend the virtual specimen manufacturing method for asphalt mixtures to asphalt mortar. Some researchers introduced the actual specimen image cutting method [16,17,18] and the random aggregate model generation method [19,20,21]. However, due to the high asphalt content and small aggregate particle size of asphalt mortar, as well as the limitation of photo pixels, the actual specimen image cutting method is difficult to apply in the manufacturing of asphalt mortar virtual specimens. Numerous researchers have chosen the random aggregate model generation method to manufacture asphalt mortar virtual specimens. Researchers have also approximated polygons as random aggregates in order to generate virtual specimens of asphalt mortar [22]. Although this method simplifies the manufacturing process of virtual specimens, there are differences between the asphalt mortar virtual specimens produced using this method and the actual asphalt mortar specimens, mainly because this method does not consider the apparent characteristics of fine aggregates in asphalt mortar.

To address the abovementioned issue, researchers first collected two-dimensional (2D) images of fine aggregates using scanning electron microscopy (SEM) [23] and then generated 2D random aggregate virtual specimens of asphalt mortar through the random aggregate placement algorithm. Although this virtual specimen manufacturing method solves the problem of aggregate apparent characteristics, several shortcomings remain, such as low efficiency in generating virtual specimens, difficulty in generating small-sized virtual specimens, and inaccurate matching between 2D aggregate area grading and three-dimensional (3D) aggregate volume grading.

Another aspect to consider is the analysis of the RVE characteristics of numerical models for asphalt mortar of different sizes. Unlike asphalt mixtures, there is little research on the RVE characteristic analysis of asphalt mortar numerical models in the literature. Theoretically speaking, geometric analysis [15,16,24] and numerical simulation [15,25] methods that are used to determine the RVE size of asphalt mixture numerical models can be applied to asphalt mortar. However, due to the lack of relevant examples, the specific applications of these analysis methods in the RVE characteristic analysis of asphalt mortar numerical models still need further development.

According to the above summary, it can be concluded that two types of defects are found in existing literature: Firstly, there is a lack of suitable virtual specimen manufacturing methods for asphalt mortar, and secondly, there is no recommended RVE size for numerical models of asphalt mortar. The present article aims to fill this research gap with two specific goals: (1) manufacturing asphalt mortar RVE that can reflect the apparent characteristics of aggregates and accurately match the 2D aggregate area gradation with the 3D aggregate volume gradation, and (2) applying the geometric analysis and numerical simulation methods in analyzing the RVE characteristics of the asphalt mortar numerical model, thus determining the minimum size of RVE of the asphalt mortar numerical model in nondestructive state. To achieve these goals, this article extends the mature virtual specimen manufacturing method for asphalt mixtures to asphalt mortar and manufactures numerical models of asphalt mortar with different sizes of RVE. Then, the characteristics of these RVEs are analyzed using geometric analysis and numerical simulation methods. Finally, the minimum size of the 2D random aggregate numerical model RVE for asphalt mortar in a nondestructive state is obtained based on the analysis results.

## 2. Manufacturing of Asphalt Mortar Numerical Model RVE

The core of determining the minimum size of an numerical model RVE is to compare the performances of different-sized numerical models and select the minimum size that meets the performance requirements. Therefore, manufacturing numerical models with different-sized RVEs is a prerequisite for determining the minimum size of the numerical model RVE.

**Figure 2:**
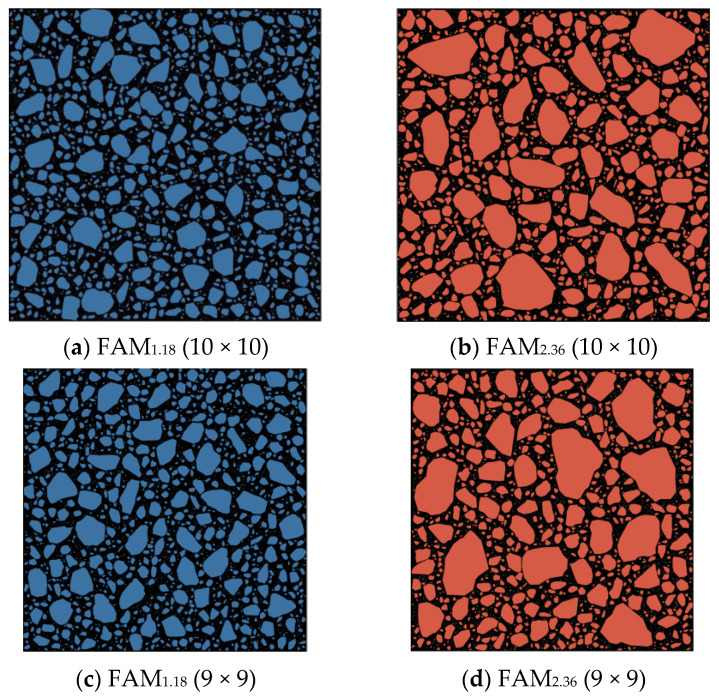

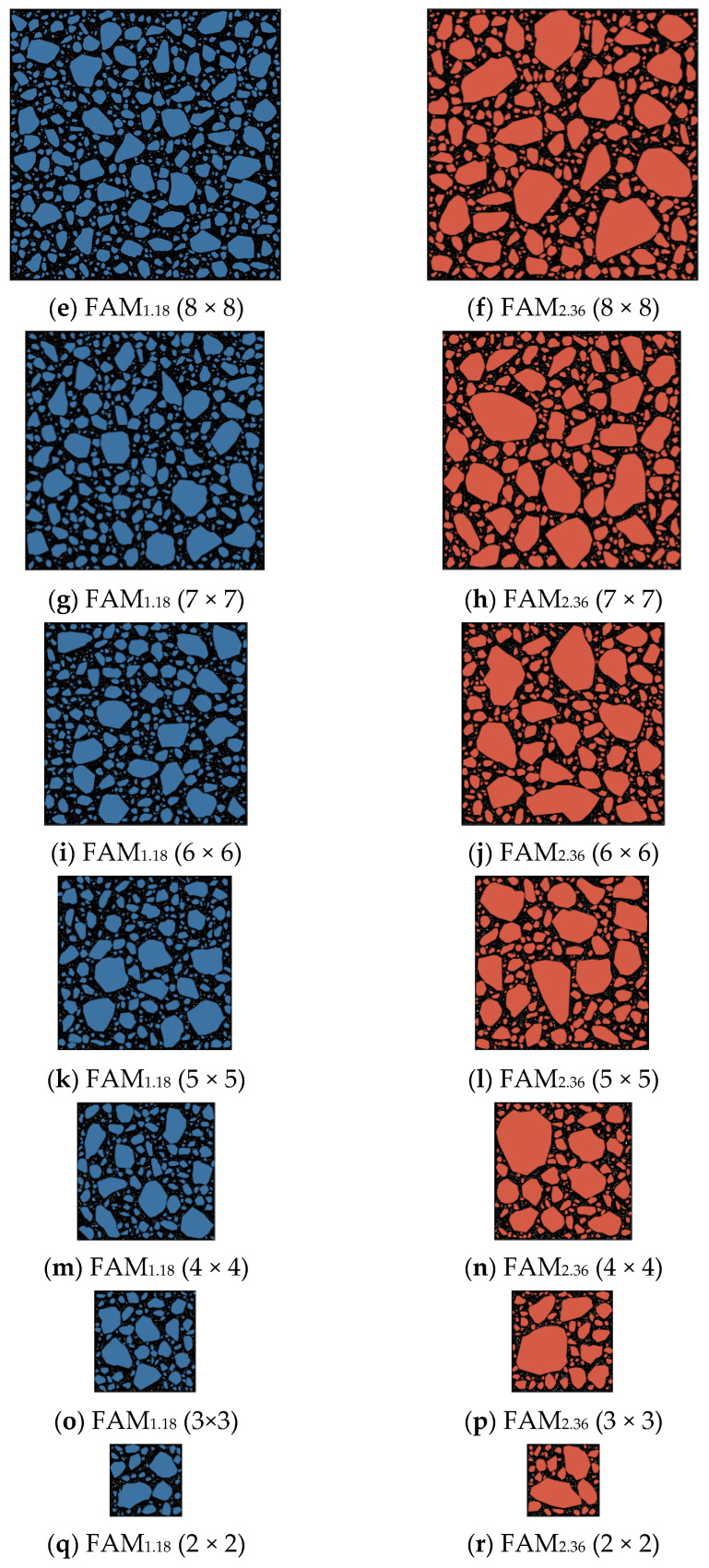
Part of virtual specimens of asphalt mortar.

**Figure 3:**
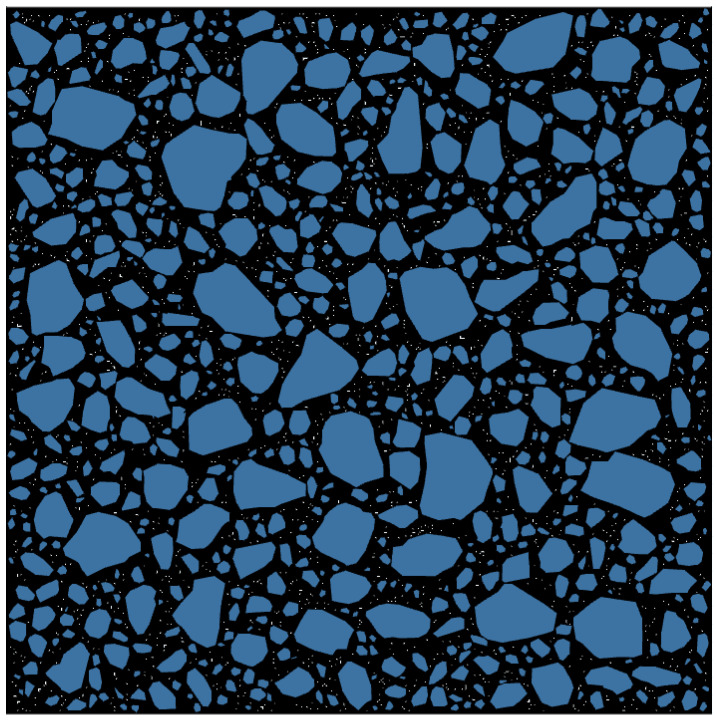
Virtual specimens of asphalt mortar: FAM_1.18_ (8 × 8).

To simplify the manufacturing process of the asphalt mortar numerical model RVE, this study sets a simplified assumption that such a model consists of fine aggregates, asphalt mastic, and voids, in which fine aggregates are aggregates with a particle size greater than 0.075 mm, and asphalt mastic is a mixture of asphalt and mineral filler with a particle size less than 0.075 mm. Based on the above assumptions, this study applied the 2D random aggregate numerical model manufacturing method for asphalt concrete to asphalt mortar [26,27] and then produced nine different-sized numerical model RVEs for two typical graded asphalt mortars. These two types of asphalt mortars correspond to AC-25C asphalt concrete with a nominal maximum particle size of 26.5 mm and maximum particle sizes of 1.18 and 2.36 mm, respectively. The gradation and void ratio are shown in Table 1. In Table 1, the gradation represents the percentage of mass passing through the sieve, and for the void ratio, the voids in the mineral aggregate (VMA) of the asphalt mixture and asphalt mortar were determined by measuring the real specimens. These nine sizes are 2 × 2, 3 × 3, 4 × 4, 5 × 5, 6 × 6, 7 × 7, 8 × 8, 9 × 9, and 10 × 10 mm, respectively. To ensure the statistical significance of the analysis, this paper set the number of parallel specimens for each size of RVE to 5, and these 5 parallel specimens have different aggregate and void distributions. A total of 90 asphalt mortar numerical models RVE were manufactured. Partial RVE generation results are shown in Figure 2. To display the components of the virtual specimen more clearly, a virtual specimen in Figure 2 is enlarged and shown in Figure 3. In Figure 2 and Figure 3, black represents asphalt mastic, white represents voids, and the rest are aggregates, and FAM_1.18_ and FAM_2.36_ represent mortar with maximum particle sizes of 1.18 and 2.36 mm, respectively.

The following steps for manufacturing asphalt mortar virtual specimens are combined with those described in the literature [27]: (1) calculate the area proportion of 2D aggregates in each particle size range, (2) generate random aggregates, (3) place random aggregates, and (4) generate random voids.

### 2.1. Calculating the Area Proportion of 2D Aggregates in Each Particle Size Range

To convert the aggregate quality grading of asphalt mortar into 2D aggregate area grading, this section used the phase separation method [26] to calculate the 2D aggregate area grading corresponding to the FAM_1.18_ and FAM_2.36_ asphalt mortar shown in Table 1. The specific steps are as follows: (1) Calculate the volume content of aggregates in asphalt mortar through the VMA of asphalt mortar; (2) Assuming the aggregate density is the same (the mass ratio of each particle size aggregate is equal to the volume ratio of each particle size aggregate), calculate the aggregate volume content of each particle size aggregate in asphalt mortar; and (3) Assuming that the VMA of asphalt mortar real specimen is equal to the VMA of 2D asphalt mortar virtual specimen, using the phase separation method [26], the aggregate volume information is converted into 2D area information, and the 2D area proportion of aggregates in each particle size range is obtained. The calculation results are shown in Table 2.

### 2.2. Generating Random Aggregates

**Figure 4:**
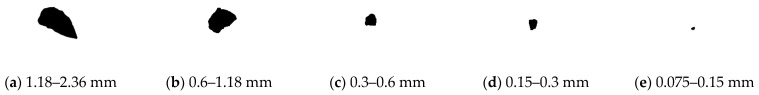
Examples of collected aggregate images for various particle size ranges.

When generating random aggregates, we first used the aggregate image testing system (AIMS) to collect targeted images of limestone aggregates with particle size ranges of 1.18–2.36, 0.6–1.18, 0.3–0.6, 0.15–0.3, and 0.075–0.15 mm. Then, using the “apparent characteristic library” method [26], we generated the required random aggregates for each particle size range. Parts of the collected 2D aggregate images are shown in Figure 4. To clearly display the outline of fine aggregate, the 2D aggregate images in Figure 4 are enlarged.

### 2.3. Placing Random Aggregates

When placing random aggregates, we estimated the number of aggregates in each grade included in the RVE of each size of asphalt mortar. The estimated results are shown in Table 3 and Table 4. Next, using the aggregate placement strategy selection method [27], we selected the appropriate aggregate placement strategy for each size of asphalt mortar RVE based on Table 3 and Table 4. Subsequently, the angle distribution of the aggregates in the asphalt mortar was set to random distribution, and the random placement method [27] was used to place the random aggregates. The following section explains the setting of aggregate angles in asphalt mortar. Unlike asphalt mixtures, asphalt mortar has a very small particle size, and the distribution of aggregate angles is not significantly affected by compaction. Therefore, to simplify the analysis, this study refers to the assumption in the literature [22,28], assuming the random distribution of aggregate angles in asphalt mortar.

### 2.4. Generating Random Voids

When generating random voids, the 2D void area ratios of FAM_1.18_ and FAM_2.36_ asphalt mortars were first calculated using the phase separation method [26]. The calculation results are shown in Table 5. Then, the minimum void was set to a square of 0.02 × 0.02 mm, and the “random pixel removal” method was used to generate random voids [26].

## 3. Geometric Analysis of the Asphalt Mortar Numerical Model RVE

Similar to the geometric analysis of RVE for asphalt mixtures described in the literature [15,16,24,27], the geometric analysis of RVE for asphalt mortar numerical models also includes 2D aggregate area proportion, aggregate angle, and RVE density uniformity analyses. This section describes the geometric analysis of the numerical model RVE for mortar. Then, a summary of the geometric analysis results is provided.

### 3.1. Analysis of the Proportion of 2D Aggregate Area

**Figure 5:**
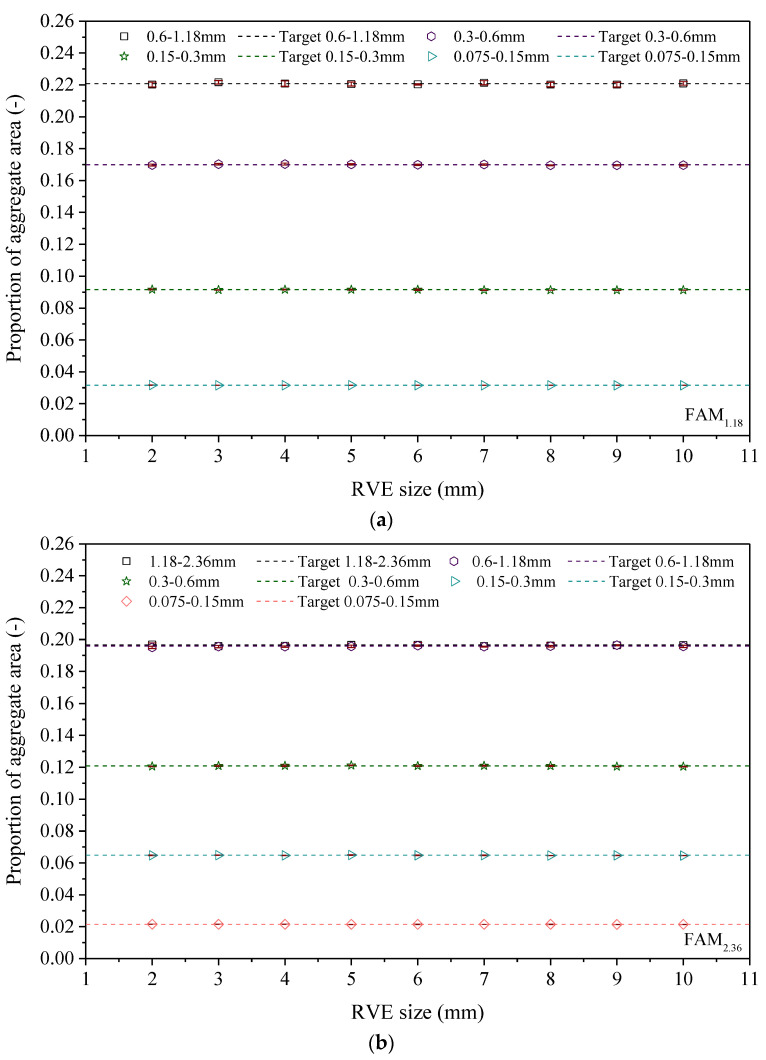

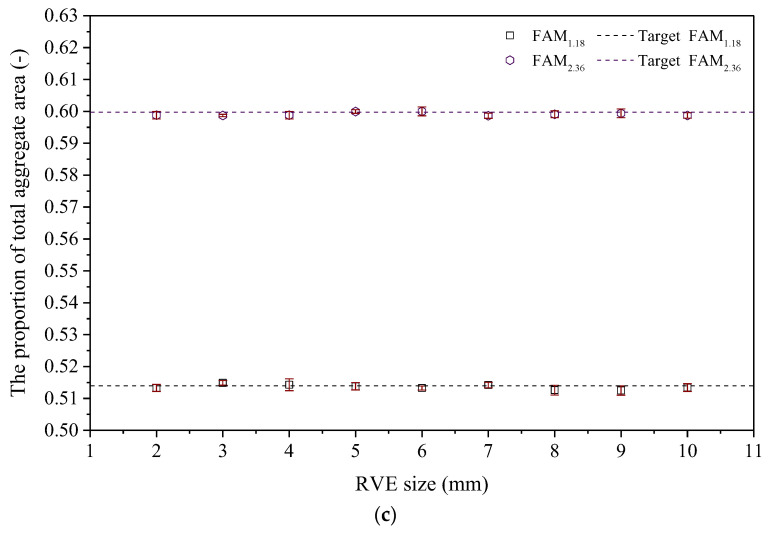
Analysis results of the proportion of 2D aggregate area. (**a**) Analysis results of the 2D aggregate area proportions in different particle size ranges of FAM_1.18_ asphalt mortar. (**b**) Analysis results of the 2D aggregate area proportions in different particle size ranges of FAM_2.36_ asphalt mortar. (**c**) Analysis results of the proportions of total areas of 2D aggregates in asphalt mortar.

Whether the proportion of 2D aggregate area in RVE is consistent with the target value is an important reference for evaluating the quality of RVE. Such proportion includes two contents: first, analysis of the proportion of 2D aggregate area in each particle size range in RVE, and second, analysis of the proportion of total aggregate area in RVE. The analysis results of the RVE area proportions of the FAM_1.18_ and FAM_2.36_ asphalt mortars manufactured in Section 2 are shown in Figure 5. Figure 5 shows the error bars representing the standard deviation. For ease of comparison, the target values for the proportion of aggregate area are also given in Figure 5.

From Figure 5a–c, we can see that in the corresponding sizes of RVE for the FAM_1.18_ and FAM_2.36_ asphalt mortars, the proportion of the 2D aggregate area is basically consistent with the target value, and the variability between parallel specimens is small. This result indicates that when the RVE size is greater than 2 × 2 mm, the proportions of 2D aggregate areas in the RVEs corresponding to the FAM_1.18_ and FAM_2.36_ asphalt mortars meet the requirements.

### 3.2. Analysis of 2D Aggregate Angles

**Figure 6:**
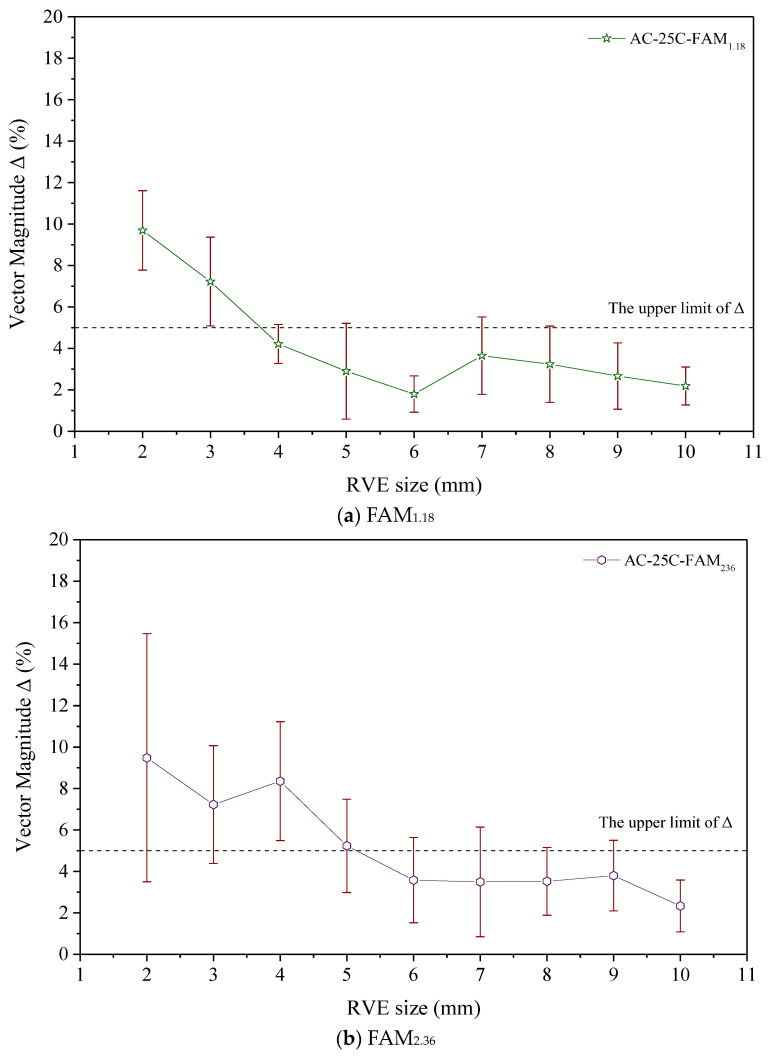
Analysis results of asphalt mortar aggregate angle.

The 2D aggregate angle can reflect the anisotropy of RVE [29]. In the current study, we referred to the relevant literature [29] and utilized Vector Magnitude Δ. The aggregate angles in the numerical model RVEs of the FAM_1.18_ and FAM_2.36_ asphalt mortars were analyzed. The analysis results with the error bars representing standard deviation are shown in Figure 6. From Figure 6a,b, it can be seen that the variability between the parallel specimens of FAM_2.36_ asphalt mortar is greater than that of FAM_1.18_ asphalt mortar. This indicates that the larger the aggregate particle size in asphalt mortar, the more difficult it is to control the distribution of aggregate angles of the virtual specimen.

To evaluate the angular distribution of RVE aggregates in various sizes of asphalt mortar, we set Δ upper limit at 5%, considering that Δ satisfies the requirement for aggregate angle distribution in RVE below 5%. When Δ = 0% represents a completely random distribution of aggregate angles, Δ = 100% indicates that the aggregate angle is exactly the same [29]. As described in Section 2.3, the distribution of aggregate angles in this study was set to a random distribution when manufacturing various sizes of RVE corresponding to asphalt mortar. Therefore, we set the target value of Δ at 0%. However, the numerical model RVE size of asphalt mortar is usually small. This poses challenges to achieving or approaching the target value of 0%. To balance the relationship between numerical calculation efficiency and 2D aggregate angle distribution, this study suggests that Δ being less than 5% is approximately random, thus meeting the requirements for aggregate angle distribution set in this study.

For ease of comparison, Figure 6 shows the upper limit of Δ. As shown in Figure 6a, when the RVE size is greater than 4 × 4 mm, the angle distribution of RVE aggregates corresponding to the FAM_1.18_ asphalt mortar meets the requirements. Similarly, as shown in Figure 6b, when the RVE size is greater than 6 × 6 mm, the angle distribution of RVE aggregates corresponding to the FAM_2.36_ asphalt mortar also meets the requirements.

### 3.3. Density Uniformity Analysis

**Figure 7:**
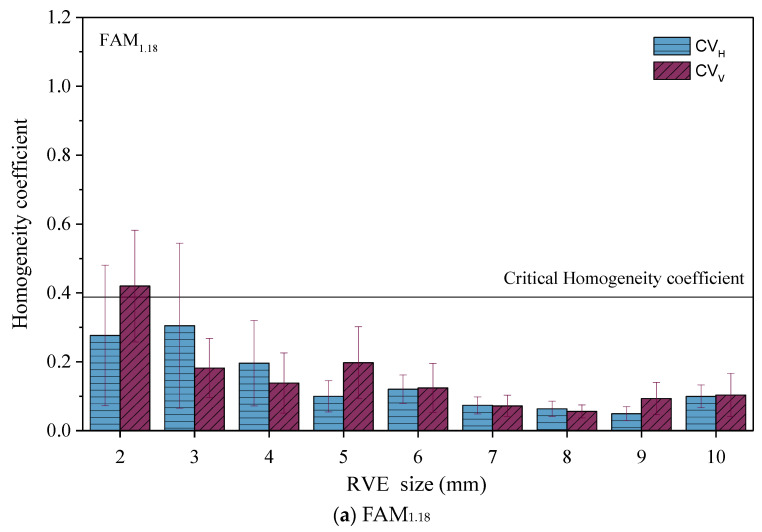

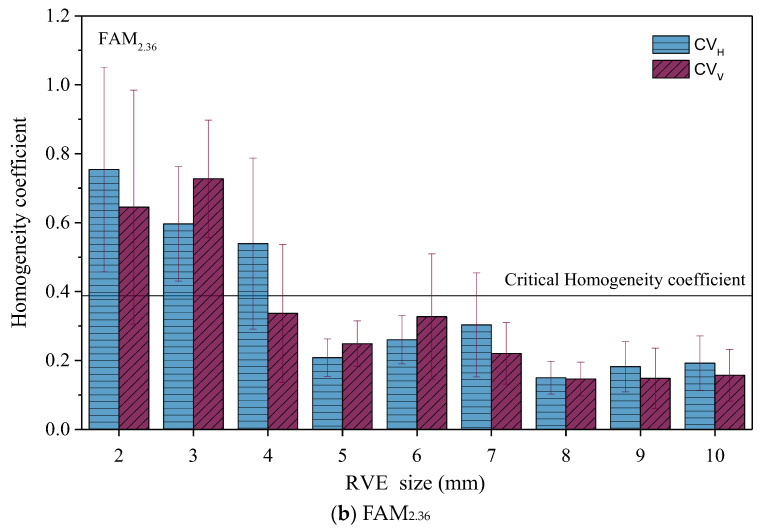
Analysis results of density uniformity of asphalt mortar.

This section refers to the literature [27] and analyzes the density uniformity of FAM_1.18_ and FAM_2.36_ asphalt mortar numerical model RVEs using the vertical density uniformity coefficient ($CV_{\rho V}$) and horizontal density uniformity coefficient ($CV_{\rho H}$). The analysis results with the error bars representing standard deviation are shown in Figure 7. The variability rule between the parallel specimens shown in Figure 7 is similar to that in Figure 6. The larger the particle size of the aggregate in asphalt mortar, the more difficult it is to control the density uniformity of the virtual specimen.

To simplify the analysis and calculation, this article assumes that the density of the same type of aggregate is the same and that the density of asphalt mastic corresponding to different gradation asphalt mortar is the same. The density set is shown in Table 6. In Table 6, the aggregate density was referenced to reference [27], and asphalt mastic density was estimated by the maximum theoretical density equation [30]. During the estimation, this study assumes that the volume proportion of filler is 60%, the volume proportion of asphalt is 40%, the effective relative density of filler is 2.7 g/cm^3^, and the relative density of asphalt is 1.034 g/cm^3^.

To evaluate the density uniformity of RVE for each size, we referred to reference [27] and calculated the critical density uniformity coefficient ($CV_{\rho S}$) for each asphalt mortar. $CV_{\rho S}$ was calculated based on Equation (1) [27].

(1) $$CV_{\rho S}=CV_{S}\left(P_{\mathrm{Agg}}\rho_{\mathrm{Agg}}+P_{M}\rho_{M}+P_{V}\rho_{V}\right)$$

In Equation (1), $CV_{S}$ is the critical coefficient of variation, and this study referred to reference [27] and set $CV_{S}$ = 0.15. $\rho_{\mathrm{Agg}}$, $\rho_{M}$, and $\rho_{V}$ are the area proportion of total aggregate, mastic, and void; and $\rho_{\mathrm{Agg}}$, $\rho_{M}$, and $\rho_{V}$ are the density of aggregate, mastic and void.

The results show that RVEs in which $CV_{\rho V}$ and $CV_{\rho H}$ are both less than $CV_{\rho S}$ meet the density uniformity requirement. Meanwhile, for the sake of comparison, we plotted the corresponding $CV_{\rho S}$ values of the FAM_1.18_ and FAM_2.36_ asphalt mortar samples in Figure 7. As shown in Figure 7, to ensure the uniformity of density, the minimum RVE dimensions corresponding to the FAM_1.18_ and FAM_2.36_ asphalt mortars should be 3 × 3 and 5 × 5 mm, respectively.

### 3.4. Summary of Geometric Analysis

This section presents the results of the RVE geometric analysis, as shown in Table 7. As shown in the table, if the RVE dimensions of FAM_1.18_ and FAM_2.36_ asphalt mortars are 4 × 4 and 6 × 6 mm or more, respectively, the RVE can simultaneously meet the requirements of 2D aggregate area proportion, 2D aggregate angle, and density uniformity.

## 4. Virtual Dynamic Compression Modulus Test of Asphalt Mortar Numerical Model RVE

In this section, we first conducted virtual dynamic compression modulus tests on 90 different sizes of RVEs corresponding to the FAM_1.18_ and FAM_2.36_ asphalt mortar samples. Then, the results of these virtual experiments were analyzed. Due to the large number of virtual specimens and the computational complexity, we have referred to a simplified scheme from literature [27] and only conducted virtual dynamic compression modulus tests at a certain temperature (10 °C) and loading frequency (10 Hz) to improve the efficiency of numerical analysis.

### 4.1. Definition of Numerical Models

**Figure 8:**
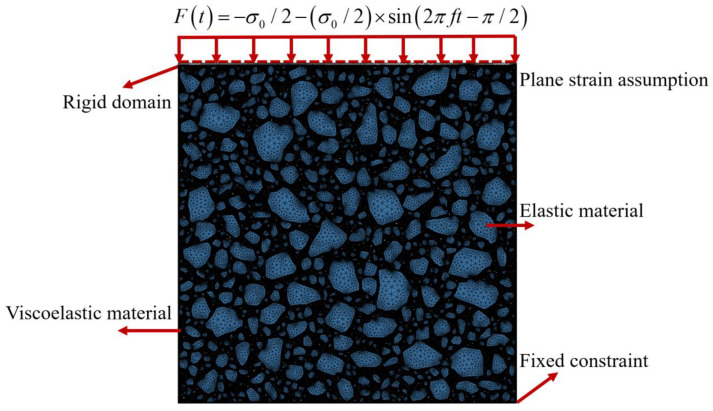
Definition of the numerical model for asphalt mortar.

In this section, we refer to the numerical model definition of asphalt mixtures [26] and define the numerical model of asphalt mortar. Combining this definition with the data shown in Figure 8, we then explain the definition of the numerical model.

In Figure 8, we assume the presence of plane strain in the numerical calculation of asphalt mortar. Meanwhile, the aggregate is defined as elastic material, and asphalt mastic is defined as linear viscoelastic properties material. The relevant material parameters are shown in Table 8 and Table 9. In particular, Table 9 refers to the Prony series parameters of the generalized Maxwell model [31]. In Table 9, *ρ*_i_ is the relaxation time of the i-th Maxwell model unit, *E*_i_ is the elastic modulus of the i-th Maxwell model unit, and *E*_e_ is the equilibrium modulus [32]. In the current work, we used a triangular mesh for mesh division, assumed the complete coordination of deformation between the interface and nodes of the aggregate and asphalt mastic elements, set a semisinusoidal load at the top of the virtual specimen, and fixed the constraints at the bottom. Additionally, we did not set friction between the aggregate and mastic, and we assumed that both sides of the virtual specimen were free boundaries.

### 4.2. Numerical Calculation and Result Processing

Here, we calculated the dynamic mechanical responses of different sizes of RVE corresponding to the FAM_1.18_ and FAM_2.36_ asphalt mortars at a temperature of 10 °C and a load frequency of 10 Hz. When conducting numerical calculations, we referred to the analysis method used in reference [27] and set the number of loading cycles to 200. A virtual experiment was conducted to select the number of calculation cycles. Then, we took the virtual dynamic compression modulus and phase angle corresponding to the 200th loading cycle as the standard values, after which we calculated the relative errors between the virtual dynamic compression modulus, phase angle, and the standard values for each loading cycle. Finally, based on the variation of relative error with the number of loading cycles, an appropriate number of loading cycles was selected. To simplify the analysis, this study suggests that calculation accuracy meets the requirements when the relative error between the dynamic compressive modulus and phase angle of asphalt mortar is less than 5%.

**Figure 9:**
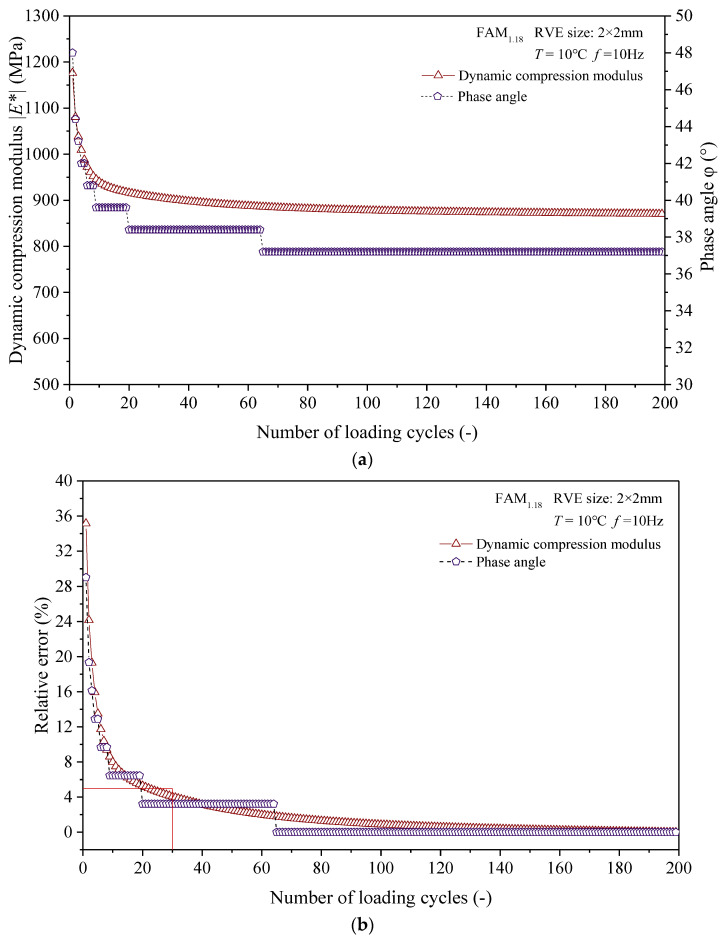
Virtual test results for determining the number of loading cycles. (**a**) Dynamic compressive modulus and phase angle vs. number of loading cycles. (**b**) Relative error vs. number of loading cycles.

The dynamic compressive modulus and phase angle of asphalt mortars obtained from the above steps as functions of the number of loading cycles are shown in Figure 9a, while the curve of relative error with the number of loading cycles is shown in Figure 9b. As shown in Figure 9b, when the number of calculation cycles is greater than or equal to 30, the relative error between the dynamic compression modulus and the phase angle is within 5%. Therefore, this study suggests that when the loading frequency is 10 Hz and the number of calculation cycles is set to 30, the calculation efficiency and accuracy of the virtual dynamic compression modulus test of asphalt mortar can be guaranteed simultaneously.

Additionally, as shown in Figure 9a,b, with the increase of loading cycles, the trend of dynamic modulus and phase angle changes is different. Below is an explanation of the reasons behind this phenomenon. The simulation value of dynamic modulus is jointly controlled by the distribution state of aggregate and asphalt mastic, the elastic modulus of aggregate, and the viscoelastic properties of asphalt mastic, while the simulation value of the phase angle is mainly controlled by the distribution state of asphalt mastic and its viscoelastic properties. Therefore, compared to the dynamic modulus, the phase angle is easier to reach a relatively stable state. When the phase angle reaches a relatively stable state, only when there is a significant change in the distribution of the asphalt mastic will the value of the phase angle change. This makes the dynamic modulus and phase angle in Figure 9 change in a different trend.

**Figure 10:**
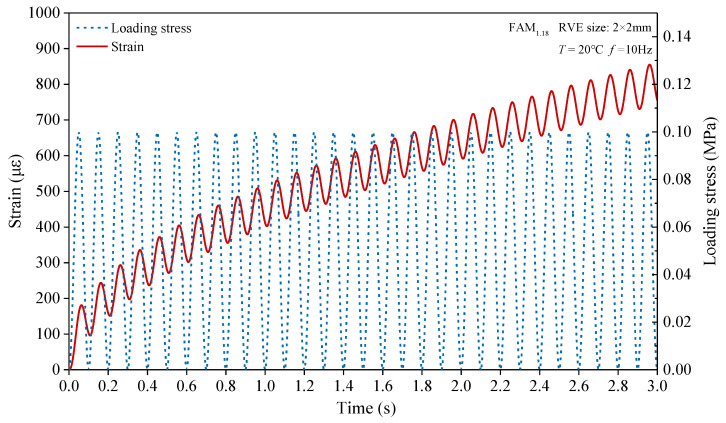
Axial stress and axial strain of asphalt mortar RVE (FAM_1.18_).

Once the numerical calculation was completed, we took a point at the top of the RVE specimen every 0.05 mm interval, derived the vertical displacement data of these points over time, and calculated the average vertical displacement of these points at each time point. Then, the average vertical displacement at each time point was divided by the original height of the RVE specimen without deformation to obtain the axial strain of the RVE specimen under uniaxial compression load, as shown in Figure 10. Next, we obtained the axial stress of the RVE specimen under uniaxial compression load based on the semisinusoidal load set by numerical experiments, as shown in Figure 10. To save space, we only present the axial stress and axial strain of a certain RVE of the FAM_1.18_ asphalt mortar, which are given as examples in Figure 10. Subsequently, relevant data processing programs were developed to calculate the dynamic compressive modulus and phase angle of RVE at each cycle based on Equations (2) and (3) [33]. Finally, we took the dynamic compression modulus and phase angle corresponding to the 30th cycle as the final result of the dynamic compression modulus and phase angle for this RVE.

(2) $$\left|E^{*}\right|=\frac{\sigma_{0}}{\varepsilon_{0}}$$

(3) $$\varphi =\frac{t_{i}}{t_{p}}\times 360$$

where $\left|E^{*}\right|$ and φ represent the dynamic compression modulus and phase angle, respectively; $\sigma_{0}$ and $\varepsilon_{0}$ represent the amplitudes of axial stress and axial strain, respectively; $t_{i}$ represents the time when the deformation peak lags behind the load peak; and $t_{p}$ represents the loading cycle.

### 4.3. Analysis of Numerical Calculation Results

**Figure 11:**
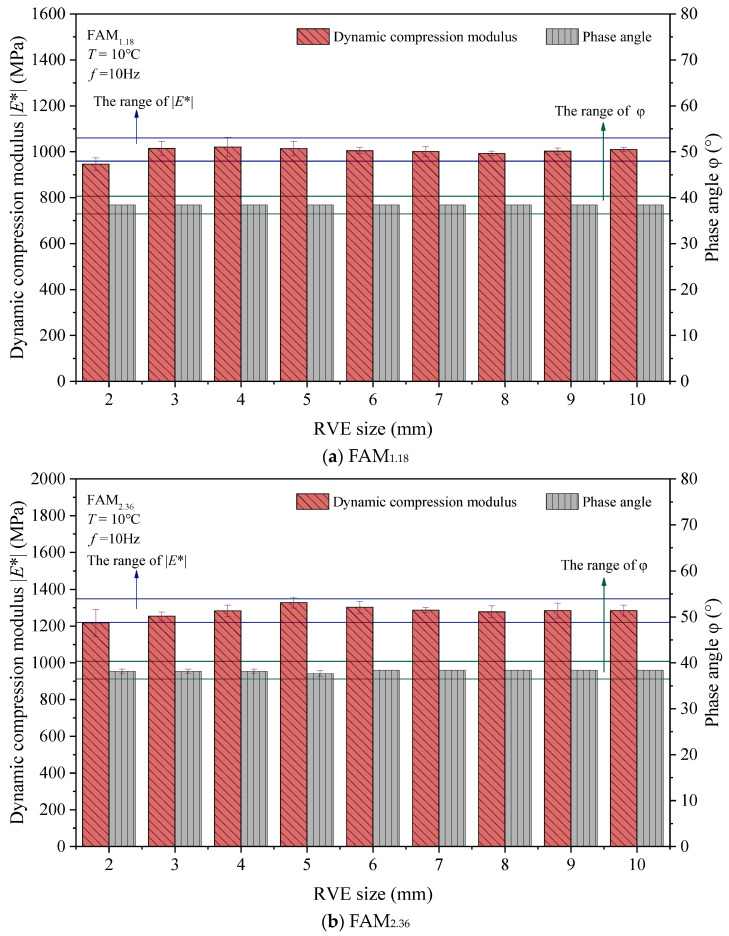
Numerical calculation results of RVE for asphalt mortar.

The calculation results of RVE for different sizes of FAM_1.18_ and FAM_2.36_ asphalt mortars are shown in Figure 11. Figure 11 also shows the error bars representing the standard deviation. From Figure 11, it can be seen that the numerical calculation results for two different maximum particle sizes of asphalt mortar have little variability, indicating that the size of aggregate particles does not significantly affect the linear viscoelastic properties of the numerical model.

To evaluate the RVE of asphalt mortars of different sizes, we take the dynamic compression modulus ${\left|E^{*}\right|}_{S}$ and phase angle $\phi_{S}$ corresponding to the RVE with a size of 10 × 10 mm as the standard values. It is known that $\left|E^{*}\right|$ is within the range of [0.95${\left|E^{*}\right|}_{S}$, 1.05${\left|E^{*}\right|}_{S}$]. Meanwhile, the RVE within the range of [0.95$\phi_{S}$, 1.05$\phi_{S}$] has a similar ability to characterize the linear viscoelastic properties of asphalt mortar with a size of 10 × 10 mm, which can be used to simulate the nondestructive viscoelastic properties of asphalt mortar. In accordance with the $\left|E^{*}\right|$ and ϕ standard ranges shown in Figure 11, it can be inferred that when the RVE size of the 2D random aggregate numerical models for the FAM_1.18_ and FAM_2.36_ asphalt mortars is 3 × 3 mm or more, the ability of RVE to characterize the linear viscoelastic properties of asphalt mortar is similar to that of RVE with a size of 10 × 10 mm.

**Figure 12:**
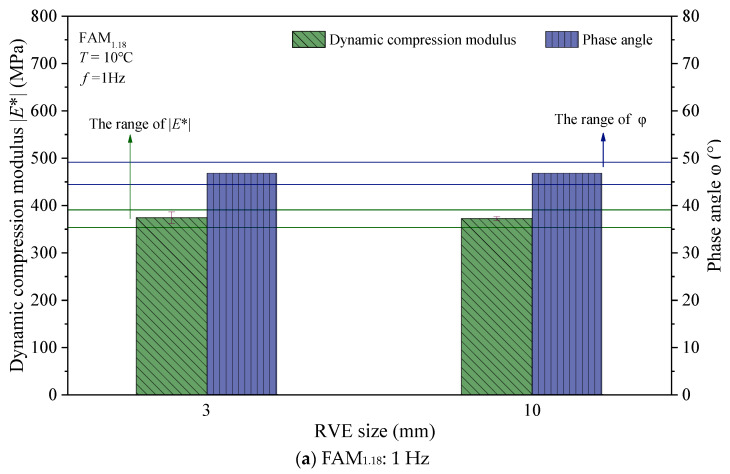

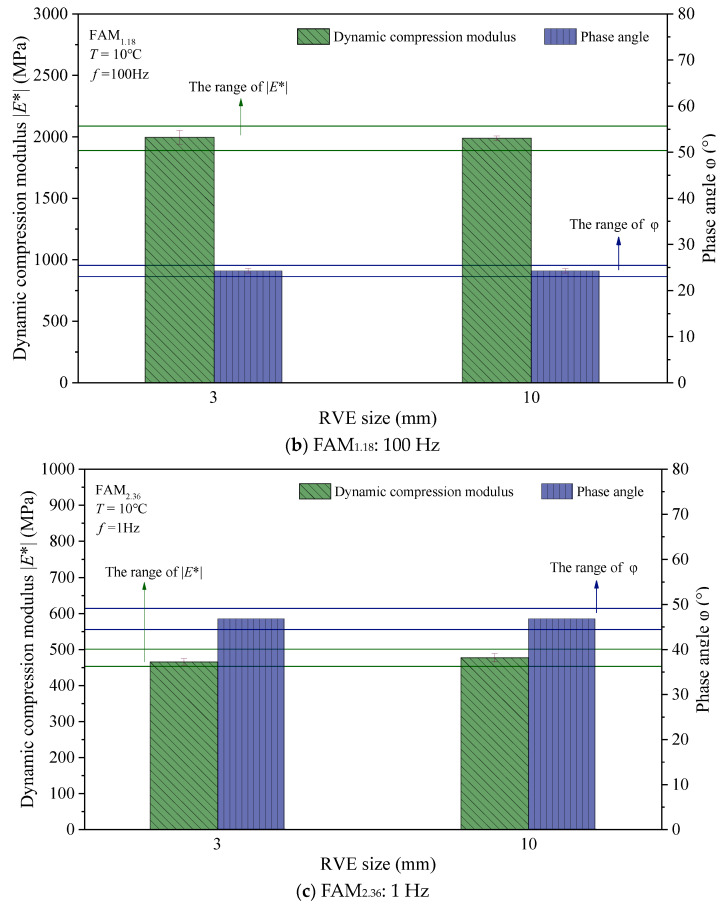

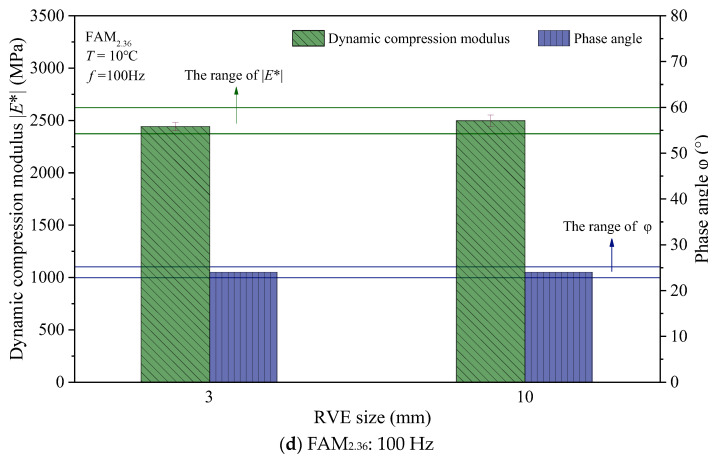
The calculation results of virtual validation test.

To examine whether the minimum RVE size obtained from virtual tests is applicable to different strain rate conditions, we designed a virtual validation test. That is, we conducted virtual tests at different loading frequencies (1 Hz, 100 Hz) to check whether the ability of RVE with a size of 3 × 3 mm to characterize the linear viscoelastic properties is similar to that of RVE with a size of 10 × 10 mm. The results with the error bars representing standard deviation for the virtual test are shown in Figure 12. Similar to Figure 11, Figure 12 also provides the standard ranges of dynamic modulus and phase angle. According to Figure 12, it can be seen that under loading frequencies of 1 Hz and 100 Hz, the asphalt mortar RVE with a size of 3 × 3 mm meets the accuracy requirements, and its ability to characterize the linear viscoelastic properties of asphalt mortar is similar to that of RVE with a size of 10 × 10 mm. This verifies that the conclusions obtained in this section are applicable to different strain rate conditions.

## 5. Discussion of Geometric Analysis and Numerical Analysis Results

To explore the laws behind the geometric and numerical analysis results of asphalt mortar, the minimum size of the numerical model RVE for asphalt mortar in a nondestructive state is given. This section discusses the correlation between the geometric and numerical analysis results, as well as between the geometric and numerical analysis methods corresponding to asphalt mortar. These discussions can be divided into two parts. First, the geometric analysis and numerical analysis results of the numerical model RVE for asphalt mortar were discussed. Then, the relationship between geometric analysis methods and numerical analysis methods was discussed. Below is a detailed description of the discussion of these two parts.

### 5.1. Geometric Analysis and Numerical Analysis Results of the Numerical Model RVE for Asphalt Mortar

This section first summarizes the geometric and numerical analysis results of asphalt mortar described in Section 3.4 and Section 4.3 and shown in Table 10. Then, the analysis results are discussed.

Based on the geometric analysis results in Table 10, the minimum RVE size of the asphalt mortar numerical model that corresponds to the geometric analysis results increases along with the increasing maximum aggregate size of the asphalt mortar. Through simple analysis, it can be concluded that this phenomenon is caused by the internal structure of RVE, in which the quality of a certain size of RVE decreases with the increase of the maximum aggregate particle size in RVE. When the maximum aggregate particle size in RVE increases, the quality of RVE can only be guaranteed by expanding the size of RVE.

Based on the analysis of the virtual dynamic compression modulus test results in Table 10, it can be seen that the minimum RVE sizes corresponding to asphalt mortar with maximum particle sizes of 1.18 and 2.36 mm are the same. Upon analyzing this phenomenon, we find that it is caused by the size setting of the RVE. In this study, nine different sizes of 2D random aggregate virtual specimens of asphalt mortar were manufactured with 1 mm intervals. Compared with asphalt mortar with a maximum particle size of 1.18 or 2.36 mm, the 1 mm interval appeared larger. This interval made it difficult to reflect the influence of the maximum aggregate particle size on the minimum RVE size. Furthermore, such an interval resulted in a more conservative minimum RVE size obtained from the analysis of virtual dynamic compression modulus test results of asphalt mortar.

### 5.2. Relationship between Geometric and Numerical Analysis Methods

This section first explains the technical paths of the geometric and numerical analysis methods. Then, the principle for selecting the minimum size of the numerical model RVE in a nondestructive state was also provided.

The geometric and numerical analysis methods are effective analytical methods for determining the minimum RVE size of asphalt mortar numerical models under nondestructive conditions, although they adopt different technical paths. On the one hand, the geometric analysis method starts from the interior of the numerical model RVE and determines the minimum size of the RVE by examining the state of aggregates, asphalt mastic, and voids inside the RVE. On the other hand, the numerical analysis method starts from the macroscopic performance of the numerical model RVE and determines the minimum size of RVE by testing whether the model can characterize the linear viscoelastic mechanical behavior of asphalt mortar. From the technical paths of these two analysis methods, it can be seen that geometric analysis is a strict test of the internal microstructure state of asphalt mortar RVE, while numerical analysis is a single test of whether asphalt mortar RVE can characterize the linear viscoelastic mechanical behavior. Therefore, compared with numerical analysis, geometric analysis is more rigorous, which is also why the results of such an analysis are significantly greater than those of numerical analysis, as shown in Table 10.

Furthermore, setting the size of RVE based on numerical analysis results can accurately characterize the linear viscoelastic mechanical behavior of asphalt mortar. In comparison, setting the size of RVE based on geometric analysis results not only accurately characterizes the linear viscoelastic mechanical behavior of asphalt mortar, but also accurately characterizes the internal microstructural changes of asphalt mortar under the linear viscoelastic mechanical behavior. Therefore, the results of numerical geometric analyses can serve as the minimum size of RVE for asphalt mortar numerical models in a nondestructive state, although their applicability varies. 

Based on the availability of computing resources and the goal of cross-scale simulation, this study provides the principle for selecting the minimum size of the numerical model RVE in a nondestructive state. In particular, if computing resources are limited, only RVE is needed to characterize the linear viscoelastic mechanical behavior of asphalt mortar. In this case, there is no longer a need to delve into the internal microstructural changes of asphalt mortar under the linear viscoelastic mechanical behavior, the numerical analysis results are prioritized as the minimum size of RVE. If computing resources are sufficient, it is necessary to deeply analyze the internal microstructural changes of asphalt mortar under the linear viscoelastic mechanical behavior while characterizing the linear viscoelastic mechanical behaviors of asphalt mortar using RVE. At the same time, the geometric analysis results must be prioritized as the minimum size of RVE.

## 6. Conclusions and Recommendations for Future Research

### 6.1. Conclusions

This article presents a method for determining the minimum size of the RVE for a 2D random aggregate numerical model of asphalt mortar in a nondestructive state. First, the manufacturing method of 2D random aggregate virtual specimens for asphalt concrete was applied to asphalt mortar, and the corresponding numerical model RVEs for the different sizes of asphalt mortars (FAM_1.18_ and FAM_2.36_) were manufactured. Then, geometric analysis was conducted on the numerical model RVE of asphalt mortar, including the 2D aggregate area proportion, 2D aggregate angle, and density uniformity analysis. Subsequently, numerical analysis was carried out on the numerical model RVE of asphalt mortar. Finally, the geometric analysis and numerical analysis results of the asphalt mortar numerical model RVE were discussed. The following conclusions have been drawn:

(1) When using RVE to characterize the linear viscoelastic mechanical behavior of asphalt mortar, it is also necessary to deeply analyze the internal microstructure changes of asphalt mortar under the linear viscoelastic mechanical behavior. The minimum size of the asphalt mortar numerical model RVE in a nondestructive state is determined by the geometric analysis results. In this case, the minimum sizes corresponding to the asphalt mortar 2D random aggregate numerical model RVE with the largest particle sizes of 1.18 and 2.36 mm are 4 × 4 and 6 × 6 mm, respectively.
(2) If RVE is used to characterize the linear viscoelastic mechanical behavior of asphalt mortar, there is no need to delve into the internal microstructural changes of asphalt mortar under the linear viscoelastic mechanical behavior. The minimum size of the asphalt mortar numerical model RVE in a nondestructive state can be determined according to the numerical analysis results. In this case, the minimum size corresponding to the 2D random aggregate numerical model RVE of asphalt mortar with maximum particle sizes of 1.18 and 2.36 mm is 3 × 3 mm.

There is no significant difference in the gradation of asphalt mortar corresponding to the dense-graded asphalt mixtures. Therefore, we believe that the conclusions presented in this study can serve as a reference to help readers choose the RVE size for asphalt mortar corresponding to the dense-graded asphalt mixtures.

### 6.2. Recommendations for Future Research

(1) The main factor affecting the size of asphalt mortar’s RVE is the size and distribution of aggregates. To simplify the analysis, this manuscript directly uses the asphalt mastic parameters from the literature. In the future, when researchers refer to the method presented in this study to determine the RVE size of asphalt mortar numerical models, they should consider conducting dynamic modulus tests on asphalt mastic, and using the Prony series parameters obtained from the test data as the material parameters to obtain simulation results that correspond to reality.
(2) This study used geometric and numerical analysis to provide the minimum size of the numerical model RVE for asphalt mortar in a non-destructive state but did not use this conclusion to conduct cross-scale simulations from asphalt mastic to asphalt mortar. In the future, relevant numerical simulations should be conducted, and the simulation results should be compared with actual experimental results to further validate the conclusions obtained in this study.
(3) Due to limited computing resources, we have referred to a simplified scheme from the literature, and only conducted virtual tests on dynamic viscoelastic properties at a certain temperature and loading frequency. In the future, when readers refer to this research method to determine the size of RVE, they should conduct dynamic viscoelastic properties virtual experiments at different temperatures and loading frequencies to further discuss the impact of different loading conditions on RVE size.

## Figures and Tables

**Table 1:** Grading and volume information of asphalt mixture and asphalt mortar.

Sieve Size (mm)	Percentage of Mass Passing Sieve (%)		
	AC-25C	FAM_2.36_	FAM_1.18_
31.5	100	—	—
26.5	99.2		
19	78.3		
16	68.4		
13.2	58.9		
9.5	46.3		
4.75	34.3		
2.36	23.1	100	
1.18	16.0	69.3	100
0.6	10.2	44.2	63.8
0.3	7.3	31.6	45.6
0.15	5.6	24.2	35.0
0.075	5.1	22.1	31.9
0	0	0	0
Asphalt content (%)	3.75	9.73	10.54
Void ratio (%)	4.1	1.59	1.19
VMA (%)	12.1	22.53	23.87

**Table 2:** Gradation and proportion of 2D aggregate area.

Sieve Size (mm)	Percentage of Area Passing Sieve (%)		Size Range (mm)	Proportion of 2D Aggregate Area (%)	
	FAM_2.36_	FAM_1.18_		FAM_2.36_	FAM_1.18_
2.36	100	-	-	-	-
1.18	74.6	100	1.18–2.36	19.7	-
0.6	49.3	71.0	0.6–1.18	19.6	22.1
0.3	33.7	48.7	0.3–0.6	12.1	17.0
0.15	25.4	36.6	0.15–0.3	6.5	9.2
0.075	22.6	32.5	0.075–0.15	2.1	3.2
0	0	0	-	-	-

**Table 3:** Estimated number of aggregates for asphalt mortar virtual specimens (FAM_1.18_).

Particle Size (mm)	Edge Length (mm)								
	10	9	8	7	6	5	4	3	2
0.6–1.18	57.3	46.4	36.7	28.1	20.6	14.3	9.2	5.2	2.3
0.3–0.6	173.1	140.2	110.8	84.8	62.3	43.3	27.7	15.6	6.9
0.15–0.3	386.5	313.1	247.4	189.4	139.2	96.6	61.8	34.8	15.5
0.075–0.15	529.2	428.6	338.7	259.3	190.5	132.3	84.7	47.6	21.2

**Table 4:** Estimated number of aggregates for asphalt mortar virtual specimens (FAM_2.36_).

Particle Size (mm)	Edge Length (mm)								
	10	9	8	7	6	5	4	3	2
1.18–2.36	12.5	10.1	8.0	6.1	4.5	3.1	2.0	1.1	0.5
0.6–1.18	50.8	41.2	32.5	24.9	18.3	12.7	8.1	4.6	2.0
0.3–0.6	123.1	99.7	78.8	60.3	44.3	30.8	19.7	11.1	4.9
0.15–0.3	273.8	221.8	175.3	134.2	98.6	68.5	43.8	24.6	11.0
0.075–0.15	360.0	291.6	230.4	176.4	129.6	90.0	57.6	32.4	14.4

**Table 5:** The proportion of 2D void area in asphalt mortar (FAM_1.18_ and FAM_2.36_).

Asphalt Mortar Type	FAM_2.36_	FAM_1.18_
Voids (%)	1.588	1.192

**Table 6:** Density settings corresponding to density uniformity analysis.

Type	$\mathrm{Aggregate}\;\mathrm{Density}\;\rho_{\mathrm{Agg}i}$ (g/cm^3^)	$\mathrm{Asphalt}\;\mathrm{Mastic}\;\mathrm{Density}\;\rho_{M}$ (g/cm^3^)	$\mathrm{Void}\;\mathrm{Density}\;\rho_{V}$ (g/cm^3^)
FAM_1.18_	2.75	2.0	0
FAM_2.36_			

**Table 7:** Summary of the geometric analysis of asphalt mortar.

Asphalt Mortar Type	Proportion of Aggregate Area	Aggregate Angle	Uniformity of Density
FAM_1.18_	2 × 2 mm	4 × 4 mm	3 × 3 mm
FAM_2.36_	2 × 2 mm	6 × 6 mm	3 × 3 mm

**Table 8:** Material parameters of aggregates and asphalt mastic.

Type	Elastic Modulus (GPa)	Poisson’s Ratio (-)	Density (g/cm^3^)
Aggregate	40	0.15	2.75
Asphalt mastic	-	0.4	2.0

**Table 9:** Prony series parameters of asphalt mastic (*T*_r_ = 10 °C) [31].

*i*	*ρ*_i_ (s)	*E*_i_ (MPa)	*i*	*ρ*_i_ (s)	*E*_i_ (MPa)
1	1.00 × 10^6^	0.02	8	1.00 × 10^−1^	81.93
2	1.00 × 10^5^	0.03	9	1.00 × 10^−2^	224.94
3	1.00 × 10^4^	0.15	10	1.00 × 10^−3^	320.27
4	1.00 × 10^3^	0.51	11	1.00 × 10^−4^	231.83
5	1.00 × 10^2^	1.86	12	1.00 × 10^−5^	218.43
6	1.00 × 10^1^	6.64	13	1.00 × 10^−6^	170.26
7	1.00 × 10^0^	23.80	*E*_e_ = 0.55 MPa		

**Table 10:** Comparison of the results.

Asphalt Mortar Type	Geometric Analysis	Analysis of Virtual Dynamic Compression Modulus Test Results
FAM_1.18_	4 × 4 mm	3 × 3 mm
FAM_2.36_	6 × 6 mm	5 × 5 mm

## Data Availability

The original contributions presented in the study are included in the article, further inquiries can be directed to the corresponding author.
